# Supercooling preservation technology in food and biological samples: a review focused on electric and magnetic field applications

**DOI:** 10.1007/s10068-020-00750-6

**Published:** 2020-07-14

**Authors:** Taiyoung Kang, Youngsang You, Soojin Jun

**Affiliations:** 1 Department of Molecular Biosciences and Bioengineering, University of Hawaii at Manoa, Honolulu, Hawaii 96822 USA; 2 Department of Human Nutrition, Food and Animal Sciences, University of Hawaii at Manoa, Honolulu, Hawaii 96822 USA

**Keywords:** Food preservation, Supercooling, Freezing, Electric and magnetic field

## Abstract

Freezing has been widely recognized as the most common process for long-term preservation of perishable foods; however, unavoidable damages associated with ice crystal formation lead to unacceptable quality losses during storage. As an alternative, supercooling preservation has a great potential to extend the shelf-life and maintain quality attributes of fresh foods without freezing damage. Investigations for the application of external electric field (EF) and magnetic field (MF) have theorized that EF and MF appear to be able to control ice nucleation by interacting with water molecules in foods and biomaterials; however, many questions remain open in terms of their roles and influences on ice nucleation with little consensus in the literature and a lack of clear understanding of the underlying mechanisms. This review is focused on understanding of ice nucleation processes and introducing the applications of EF and MF for preservation of food and biological materials.

## Introduction

The majority of foods are susceptible to spoilage due to mainly the growth of pathogenic bacteria and the enzymatic activities during storage. It is estimated that approximately 25–30% of perishable food is wasted because of the food spoilage and most of this wastage could be saved if stored properly (Stonehouse and Evans, 2015). Numerous intrinsic and extrinsic factors such as moisture content, pH, the presence of enzymes, oxygen concentration, and exposure to light are associated with the rate of food spoilage, but it is well agreed on that temperature management is the most effective way to retard bacterial growth and slow chemical reactions (Atlas, 2006; Jakobsen and Bertelsen, 2000; Pérez-Rodríguez et al., 2018). Refrigeration and freezing (i.e. cold storage) are the oldest and most widely used methods of preservation. Refrigeration is the process in which heat is removed from foods that present at a higher temperature than its surrounding environment. In general, commercial and household refrigerators are operated at below 7 °C or a slightly lower temperature above the freezing point of foods. Refrigeration effectively delays spoilage of perishable foods by decreasing the chemical and biological processes; however, it will only extend the shelf-life for a few days or weeks at the most depending upon the type of product. By lowering the temperature, water within a food material is inevitably converted to a solid state. Freezing is recognized as the most popular method currently available for preserving foods and biological materials for an extended period. Nevertheless, the formation of ice crystals irreversibly affects the microstructure of the frozen foods because the specific volume of ice is substantially greater than that of water, thereby ice crystals compress the food matrix and cause the undesirable release of exudate after thawing (Evans, 2009). Furthermore, frozen storage leads to an increase in the concentration of solutes in the unfrozen phase, which is the main reason responsible for the quality deterioration of frozen foods such as protein denaturation, lipid oxidation, and degradation of color, pigment, and flavor (Leygonie et al., 2012; Zaritzky, 2006). Thus, a new technique based on subzero nonfreezing preservation has been of interest to researchers. One technique that addresses the problems associated with freezing is known as supercooling.

Supercooling is defined as the process of lowering the temperature of a food material below its equilibrium freezing point without the formation of ice crystals (Stonehouse and Evans, 2015). There has been continuous interest in applications of supercooling technology for food preservation since it promises an extended shelf-life while avoiding ice crystal formation and maintaining fresh textural integrity (Shafel, 2015; Stonehouse and Evans, 2015). Previous research has shown the clear benefits of supercooling preservation over the conventional cold storage methods; however, unfortunately, the supercooled state is highly unstable and ice nucleation is induced by a stochastic process, leading to the difficulty in achieving the technical stability and reproducible results (Deora et al., 2013; James et al., 2009). Moreover, since the supercooled state within foods has been mostly achieved through strict temperature control, it is extremely challenging to maintain the supercooled state for a period sufficiently long when the products are subjected to external influences such as physical vibration and temperature fluctuations (Fukuma et al., 2012; Wilson et al., 2003).

External electric field (EF) and magnetic field (MF) appear to influence the onset of ice crystal formation during supercooling since water consists of dipole molecules and is also a diamagnetic substance (Woo and Mujumdar, 2010). Water molecules naturally present in foods tend to realign and re-orientate by the applications of EF and MF either in tandem with or separately. This behavior indicates that EF and MF are potentially able to inhibit ice nucleation and may lead to a substantial change in the supercooling behavior of food products. However, the effects of EF and MF on the nucleation process remain a highly controversial issue because the underlying mechanisms are not fully elucidated and investigations into wide ranges of intensities, frequencies, exposure times, etc. are unexplored (Dalvi-Isfahan et al., 2017b; Otero et al., 2016). Moreover, the process of ice nucleation is perceived as an enigmatic phenomenon due to the spontaneous and stochastic nature (Hartmann et al., 2013). Therefore, further and deeper investigations are needed to draw meaningful conclusions about the effects of EF and MF on the control of ice nucleation and the extension of supercooling. In this review, the concepts of ice nucleation and its significance on the extension of supercooling are addressed. Additionally, the current literature on the applications of EF and MF for controlling the nucleation processes are also reviewed.

## Ice nucleation and supercooling in biological systems

### Ice nucleation: the first step toward freezing

Nucleation is the process of the creation of a new thermodynamic phase from an existing phase with high free energy to an ordered structure with low free energy. In freezing, ice nucleation is the key step in determining the overall freezing characteristics and the quality of final products (Petzold and Aguilera, 2009). There are abundant fundamental and theoretical approaches have been proposed such as phenomenological, kinetic, and microscopic analyses to properly describe and quantify the nucleation events; however, it still remains a difficult task because ice nucleation occurs at the molecular level with very short time scales (Karthika et al., 2016). Although limitations of classical nucleation theory (CNT) are inherent, it is considered as the most common theoretical framework and offers a basic platform to predict a temperature-dependent nucleation rate via thermodynamic and kinetic components. In most cases, ice nucleation in a supercooled liquid occurs by homogeneous and heterogeneous (Kiani and Sun, 2011). Homogeneous nucleation takes place in homogenous particle-free supercooled liquids when thermal fluctuations in the molecular arrangement can lead to the spontaneous formation of a stable structure that serves as the critical nucleus without the involvement of foreign substances. Homogeneous nucleation rarely occurs in most systems because it requires very large supercooling degrees; however, it is basically taken into consideration in theoretical approaches due to the complexities of nucleation (Karthika et al., 2016). The Gibbs free energy of a spherical crystallite (new phase, $\Delta G_{n}$) in mother phase (supercooled liquid) can be described by the sum of the surface free energy ($\Delta G_{S} )$ and the volumetric free energy ($\Delta G_{V} )$:

1 $$\Delta G_{n} = \Delta G_{S} + \Delta G_{V}$$

**Fig. 1:**
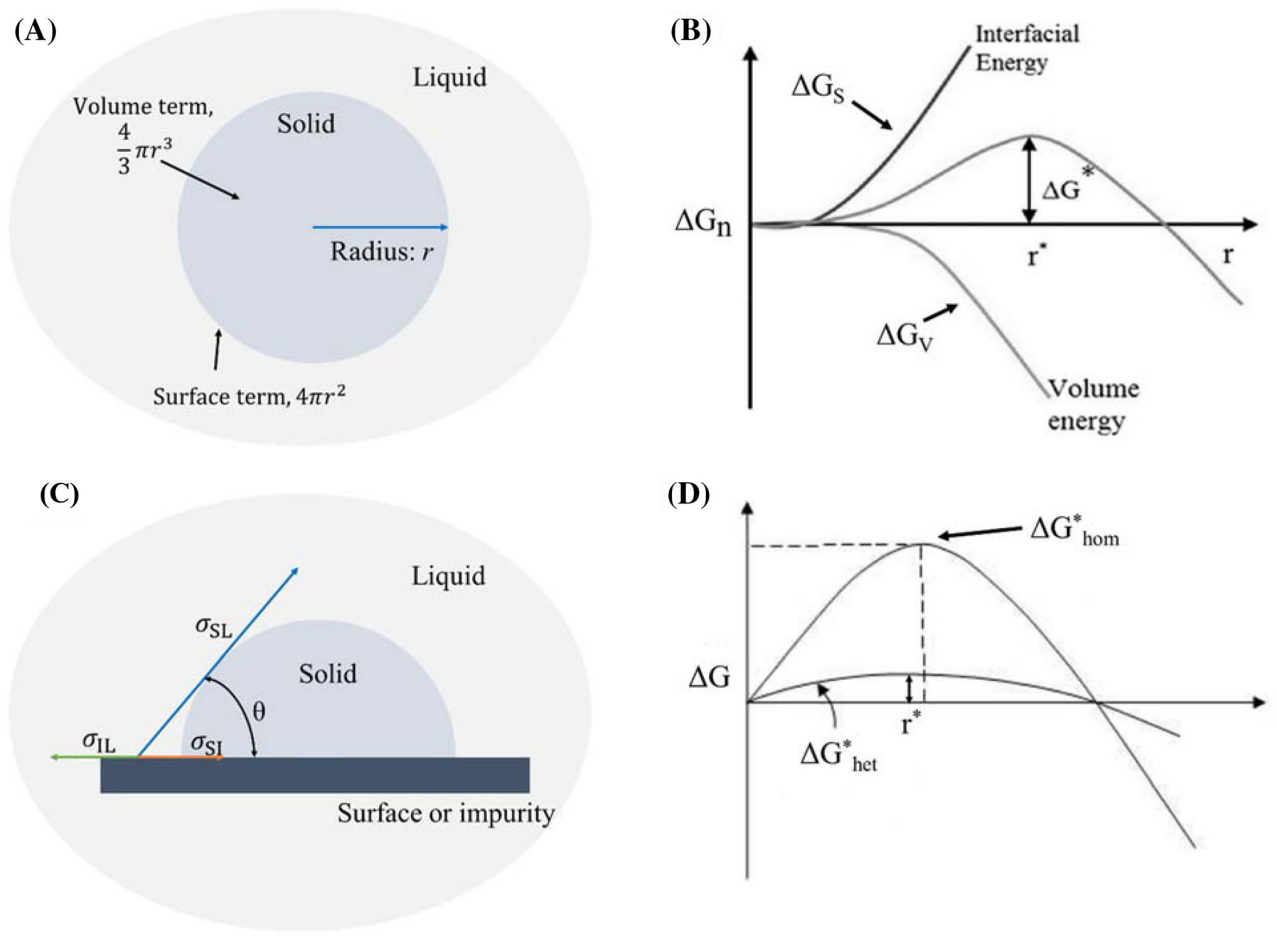
(**A**) Homogeneous nucleation. (**B**) Changes in the free energy of a spherical nucleus of radius *r* during homogeneous nucleation based on the CNT (Karthika et al., 2016). (**C**) Heterogeneous nucleation on a surface. (**D**) Comparative representation of the Gibbs free energy for homogeneous ($\Delta {\text{G}}_{ \hom }^{ *}$) and heterogeneous ($\Delta {\text{G}}_{\text{het}}^{ *}$) (Çelikbilek et al., 2012)

The change in the free energy of a spherical nucleus of radius *r* during homogeneous nucleation (Fig. 1A) is given by:

2 $$\Delta G_{n} = 4\pi r^{2} \sigma - \frac{4}{3}\pi r^{3} \Delta G_{V}$$

where $\sigma$ is the surface energy of the particle per unit volume. The first and second term of Eq. 2 presents the increase in energy required to create a new surface and the bulk free energy, respectively (Ickes et al., 2015; Kiani and Sun, 2011). Equation 2 indicates that the change in the Gibbs free energy depends upon the radius of the nucleus. For the nucleus of smaller *r,* the first term dominates, leading to the increase in the free energy; while the second term governs when *r* is larger, which leads to the overall decrease in free energy (Karthika et al., 2016). The critical radius ($r^{*}$) is the radius at which the Gibbs free energy curve reaches at the maximum value (Fig. 1B, $\Delta G^{*} )$ and it can be obtained by differentiating Eq. 2 with respect to *r*:

3 $$r^{*} = 0 = 8\pi r\sigma - 4\pi r^{2} \Delta G_{V} = - \frac{2\sigma }{{\Delta G_{V} }}$$

Then, the critical free energy ($\Delta G_{n}^{*}$) in which the energy barrier to overcome is written by:

4 $$\Delta G_{n}^{*} = \frac{16}{3} \frac{{\pi \sigma^{3} }}{{\Delta G_{V}^{2} }}$$

Since water clusters smaller than a critical size are unstable, they spontaneously appear and disappear through thermal fluctuations. However, once a cluster reaches a critical size (ice embryo), the additional inclusion of water molecules gradually lowers the free energy and the ice embryo continues to grow, become thermodynamically favored, and towards crystallization (Moore and Molinero, 2011). Supercooling acts as a driving force to overcome the free energy and the magnitude of the force is proportional to the degree of supercooling (Kiani et al., 2011). The difference between the Gibbs free energy of liquid and solid is proportional to the supercooling below the melting point, then $\Delta G_{V}$ can be rewritten as:

5 $$\Delta G_{V} = \frac{{\Delta H_{f} \Delta T }}{{T_{m} }}$$

where $$\Delta H_{f}$$ is the enthalpy of fusion, $$T_{m}$$ is the melting point, $$\Delta T$$ is the degree of supercooling ($$T_{m} - T)$$. Thus, Eq. 4 can be expressed by:

6 $$\Delta G_{n}^{*} = \frac{16}{3} \left( {\frac{{\pi \sigma^{3} T_{m}^{2} }}{{\Delta H_{f}^{2} }}} \right)\frac{1}{{\Delta T^{2} }}$$

Equation 6 implies that the high degree of supercooling decreases both the critical radius size and the free energy, resulting in an increasing rate of nucleation. The number of ice nuclei (*N*) per unit volume of a supercooled liquid is expressed by an Arrhenius type relationship (Ickes et al., 2015; Kiani and Sun, 2011):

7 $$N \left[ {{\text{m}}^{ - 3} } \right] = N_{1} { \exp }\left( {\frac{{ - \Delta G_{n} }}{{k_{B} T}}} \right)$$

where $N_{1}$ is the volume-based number density of water molecules in the liquid phase, $k_{B}$ is the Boltzmann constant. In the CNT, the nucleation rate ($J$) is defined as the formation rate of the critical ice nucleus in a unit volume of water over time. It is given by combining the thermodynamic and the kinetic terms at the steady-state:

8 $$J \left[ {{\text{m}}^{ - 3} {\text{s}}^{ - 1} } \right] = Zf^{*} {\text{C}}_{0} \exp \left( { - \frac{{\Delta G_{n}^{*} }}{{k_{B} T}}} \right)$$

where *Z* is the Zeldovich factor, $f^{*}$ is the frequency of monomer attachment to the nucleus, ${\text{C}}_{0}$ is the concentration of nucleation sites (Kiani and Sun, 2011). The kinetic part in Eq. 8 ($Zf^{*} {\text{C}}_{0}$) is related to the flux of water molecules available to transfer into the critical ice nucleus and it can be stated as the diffusive flux, the nucleus surface characteristics, and the kinetic prefactor (Ickes et al., 2015; Li et al., 2011). In biological systems, ice nucleation in a supercooled liquid is always heterogeneous (Wilson et al., 2003). In heterogeneous situation, ice nucleation is catalyzed by structural inhomogeneities such as container surfaces, dislocations, and any solid or liquid impurities in contact with water. These foreign phases significantly reduce the surface free energy of the nuclei and consequently lower the energy barrier. Figure 1(C) shows a schematic of heterogeneous nucleation on surface material and $\sigma_{\text{IL}}$, $\sigma_{\text{SL}}$, $\sigma_{\text{SI}}$ denotes the interfacial energy of liquid-surface, solid–liquid, and solid-surface by vectors, respectively. Balancing the interfacial energy in the plane of the surface wall can be expressed by:

9 $$\sigma_{\text{IL}} = \sigma_{\text{SI}} + \sigma_{\text{SL}} {\text{cos }}\uptheta$$

where $\uptheta$ is the wetting angle between $\sigma_{\text{SL}}$ and $\sigma_{\text{SI}}$. Thus, the critical radius (Eq. 3) and the critical free energy (Eq. 4) in heterogeneous nucleation situation can be expressed by:

10 $$r^{*} = - \frac{{2\sigma_{\text{SL}} }}{{\Delta G_{V} }}$$

11 $$\Delta G_{n}^{*} = \frac{16}{3} \frac{{\pi \sigma_{\text{SL}}^{3} }}{{\Delta G_{V}^{2} }} \left\{ {\frac{{\left( {2 + { \cos }\uptheta} \right)\left( {1 - { \cos }\uptheta} \right)^{2} }}{4}} \right\}$$

Equations 10 and 11 indicate that the critical radius for heterogeneous nucleation doesn’t change; whereas the activation energy barrier becomes much smaller than that of homogeneous nucleation (Fig. 1D). This statement implies that ice nucleation takes place much easier and faster in heterogeneous nucleation and the probability of supercooling extension becomes lower (Kiani and Sun, 2011). Thus, heterogeneous nucleation is preferentially observed at significantly higher temperatures compared to homogeneous nucleation (Morris and Acton, 2013). As has been shown, ice nucleation depends upon many factors such as temperature (Murray et al., 2010; Vali, 1971), freezing rate (Heneghan et al., 2001; Vali, 1994), the presence of impurity (Chen et al., 1998; Heneghan et al., 2002; Kobayashi et al., 2016), volume of sample (Kubota et al., 1988), the presence of a water/vapor interface (Li et al., 2013), viscosity (James, 1985). Particularly, supercooling is the most critical parameter in determining the nucleation rate and the probability of occurrence of the critical ice embryos (Stöckel et al., 2005; Sun, 2016; Vali and Stansbury, 1966; Wilson et al., 2003). As stated, it is problematic to accurately predict nucleation events in biological systems and even pure materials since nucleation is extremely sensitive to small inaccuracies in thermodynamic data such as density, surface tension, or pressure (Vehkamäki, 2006) and the stochastic nature of ice nucleation leads to non-reproducible results. In practice, novel techniques that control the stochastic nature of ice nucleation into a repeatable and manageable manner appear potentially beneficial for preserving foods and biological substrates such as cells, tissues, and organs during freezing processes (Morris and Acton, 2013; Petzold and Aguilera, 2009; Xanthakis et al., 2014a).

## Importance of supercooling in the freezing process

**Fig. 2:**
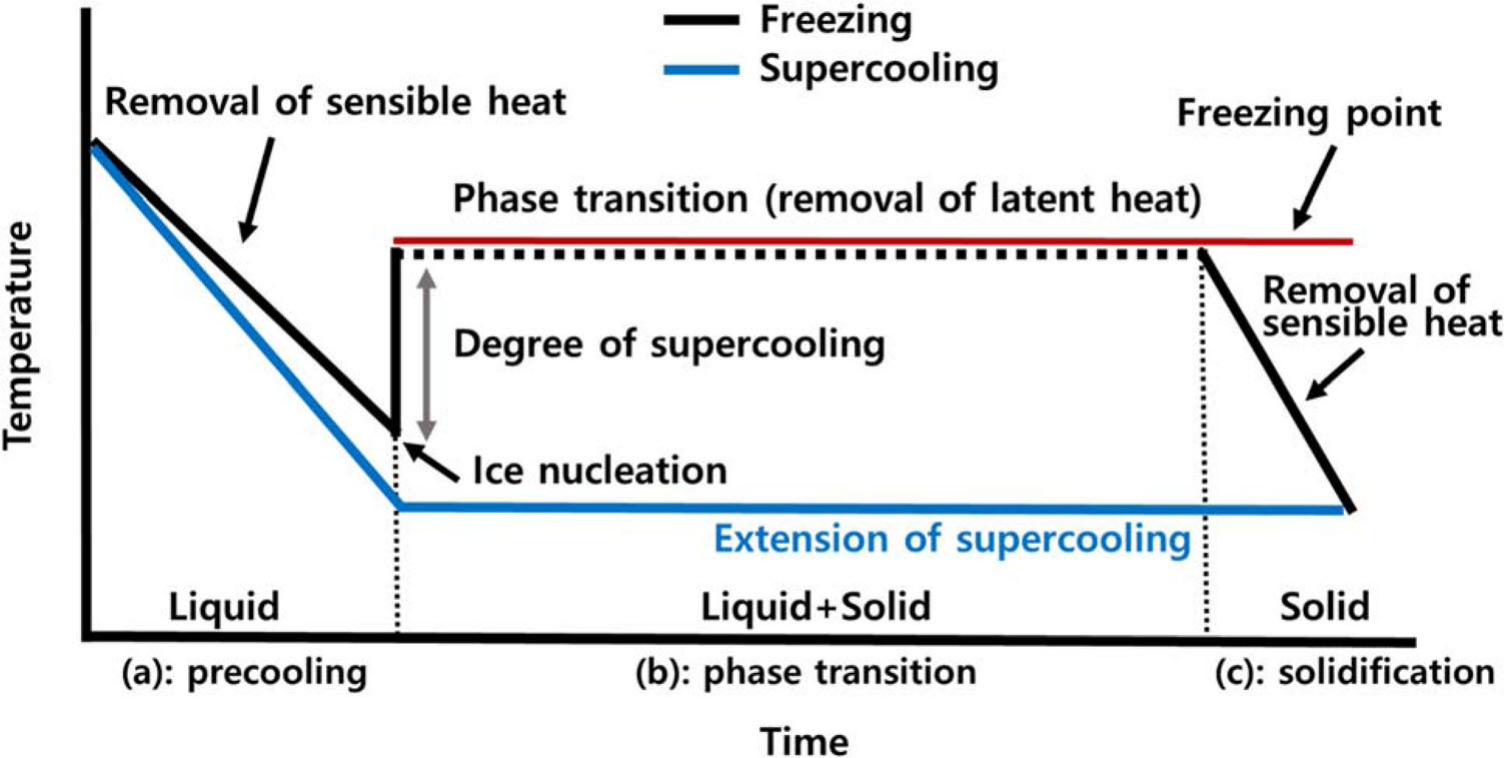
Typical time–temperature profiles of water during freezing and supercooling processes

Freezing has been known to be an extremely effective method and can possibly provide a high degree of safety, sensory quality, and even nutritional value (Evans, 2009). Figure 2 presents a typical temperature profile of water during freezing and supercooling. In general, the freezing involves three stages; (a): precooling to the freezing point, (b): phase transition (removing the latent heat, 334 kJ/kg for pure free water), and (c): further cooling to the final storage temperature. During the stage (a), the temperature of water decreases to the freezing point (ideally, 0 °C) as removing the sensible heat. Below the freezing point, water remains in the liquid phase with a certain degree of supercooling and once ice nucleation occurs, ice crystals begin to form. The temperature of the water remains at the freezing point until the phase transition is completed (b). When all the liquid water is converted into solid ice, the temperature of the ice rapidly decreased as sensible heat is removed (c) (Evans, 2009; Sun, 2016). In the past few decades, there have been considerable interests in the development of emerging technologies that suppress or promote the degree of supercooling by controlling ice nucleation. The degree of supercooling is defined as the difference between the equilibrium freezing point (red line in Fig. 2) and the temperature at which ice nucleation firstly occurs. During freezing, the suppression of supercooling may produce larger ice crystals, which has some beneficial effects in freeze-drying and freeze concentration processes (Gavish et al., 1990; Nakagawa et al., 2006; Searles et al., 2001). On the other hand, the promotion of the degree of supercooling during the freezing process will produce small and fine ice crystals throughout foods and biological substances, resulting in enhancing the quality of frozen products (Li and Sun, 2002; Teraoka et al., 2002; Zhu et al., 2005). The novel and innovative technologies for controlling the degree of supercooling have been widely researched and investigated such as high-pressure, ultrasound irradiation, and electromagnetic field (EMF)-assisted freezing. The concepts and mechanisms have been comprehensively reviewed with clarity by several authors (Cheng et al., 2017; Dalvi-Isfahan et al., 2017b; James et al., 2015b; Morris and Acton, 2013).

## Supercooling as a novel foundation of chill preservation

Supercooling is a metastable state in which the temperature of a cooled material drops down below its freezing point without ice crystal formation. This novel cooling process has been named differently such as subcooling, undercooling, and freezing point depression (Stonehouse and Evans, 2015). In this state, foods and biological substances remain unfrozen below subzero temperatures such as − 5 °C, indicating that the products that are preserved in the supercooled state do not involve in the removal of the latent heat and the phase transitions (blue line in Fig. 2). This fact implies that the supercooling preservation enables to extend the storage life of perishable products while avoiding freezing damage which is caused by ice crystal growth. Furthermore, supercooling is the potential to be an energy-efficient technology since the energy required for the freezing and thawing process is not necessary (Leadley, 2015). At the atmospheric pressure, small water droplets (micrometer-size) can be supercooled below − 40 °C; however, unfortunately, it seems very difficult to create and maintain supercooling within foods and biological substances since the supercooled state is thermodynamically unstable and the nucleation phenomenon is frequently unpredictable due to the random nature (Stonehouse and Evans, 2015). In addition to that, the structure of a solid material provides surfaces for the initiation of ice crystals (Yin et al., 2005) and several internal and external factors such as bacterial ice nucleators (Li and Lee, 1995) and physical shocks (Akio et al., 1992) also contribute to heterogeneous ice nucleation. Thus, the observed supercooling temperature in foods and biomaterials is much higher than in pure water (James et al., 2009; Jeremiah and Gibson, 1997).

In the literature, the most common approach to achieve supercooling within foods and biomaterials has been precise and strict temperature control. For example, rat livers were successfully supercooled under a stepwise cooling method (Berendsen et al., 2014; Bruinsma et al., 2015). Similarly, Usta et al. (2013) preserved rat hepatocytes in the supercooled state at − 4 °C for a week and Sultana et al. (2018) acquired supercooling within rat kidneys at − 2 °C and − 5 °C under “controlled preservation conditions”. Fuller and Wisniewski (1998) demonstrated that a deep supercooling (− 8 °C) could be achieved within cauliflower in case the surface of the vegetative plant was not covered in water under the controlled temperature condition. During ice slurry production, the accurate temperature control allowed water to be supercooled before crystallization (Bédécarrats et al., 2010). James et al. (2009) reported that peeled garlic cloves were stored at the temperature between − 6 and − 9 °C without ice crystal formation under static air conditions. The research group also tested several types of vegetables such as broccoli, carrot, cauliflower, garlic, leek, parsnip, and shallot. They reported that all replicates of garlic and shallot samples were supercooled while only 40% of parsnip showed the supercooling phenomenon. In particular, unpeeled shallots were stored at the temperature at around − 6 °C for 24 h without ice nucleation (James et al., 2011). Stepwise cooling processes (lowering 1 °C and 0.5 °C per day) were applied to achieve supercooling within fish meat below − 5 °C (Fukuma et al., 2012) and it was reported that the slow cooling rates caused a high degree of supercooling in rice starch gels (Charoenrein and Preechathammawong, 2010). Meanwhile, Choi et al. (2017) stored *Dongchimi* (a watery variety of kimchi) in “the supercooled chamber” at the set temperature of − 3.5 ± 0.5 °C with UV irradiation treatment for 15 days. They showed that the supercooling preservation at − 3 °C effectively delayed the growth of microorganisms. Another point of view on supercooling was suggested from the same institute. Choi et al. (2019) evaluated the possibility of supercooling for the purpose of cold adaptation of a kimchi starter prior to freeze-drying and reported the supercooling significantly increased the storage stability. The studies focused on precise and strict temperature control with a programmed cooling protocol at a certain cooling rate may be possible to maintain a supercooled state with no advanced technologies; however, oftentimes, the detailed cooling protocols have not been specifically described and the supercooling stability and reproducibility are still questionable.

## Emerging technologies for extended supercooling

**Fig. 3:**
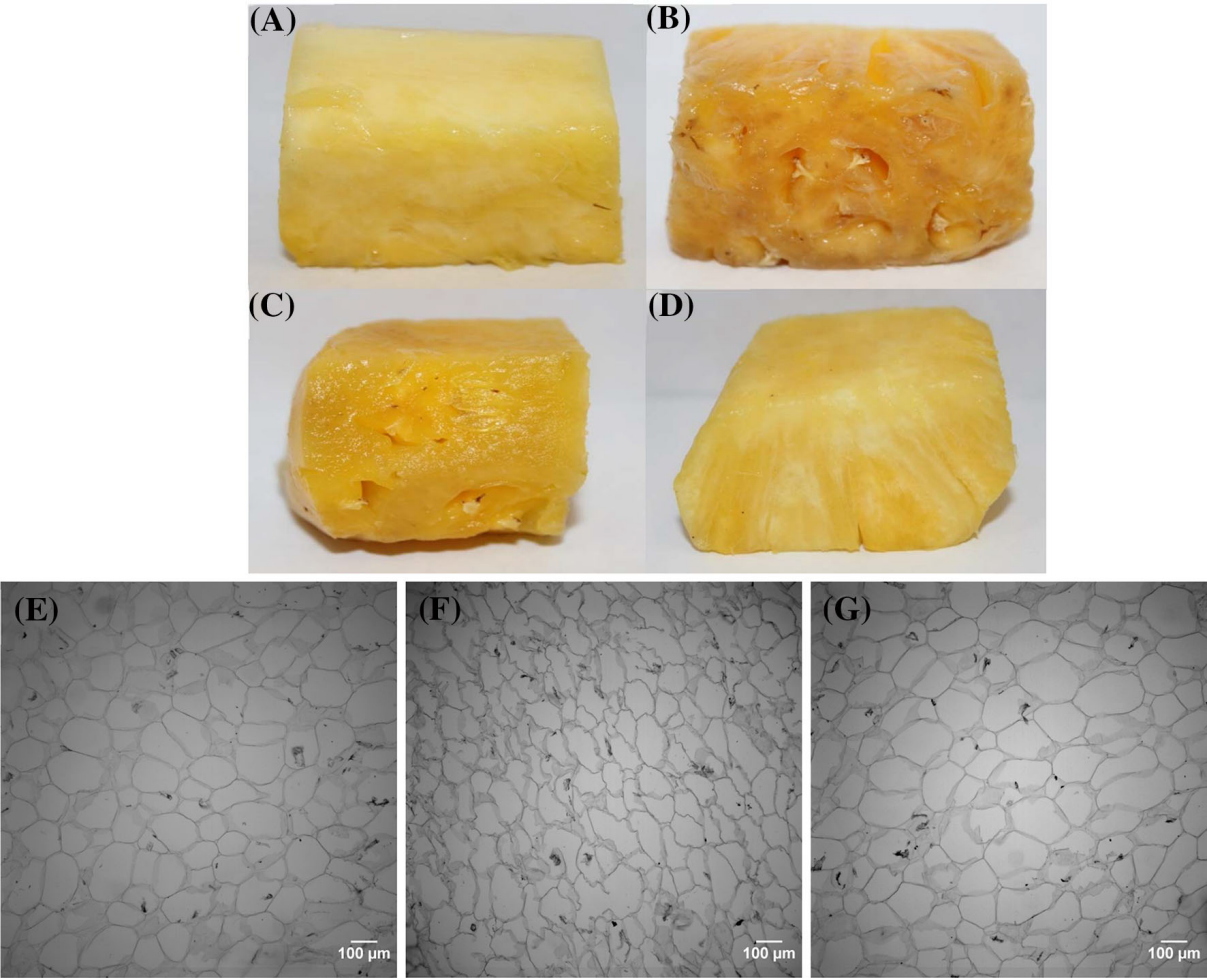
Color differences (**A**–**D**) and microstructure images (**E**–**G**) of fresh-cut pineapples preserved at different storage conditions (refrigeration: 4 °C, freezing: − 18 °C, and supercooling: − 7 °C) for 14 days: fresh (**A**, **E**), refrigeration (**B**), freezing (**C**, **F**), and supercooling (**D**, **G**) (Kang et al., 2019)

**Fig. 4:**
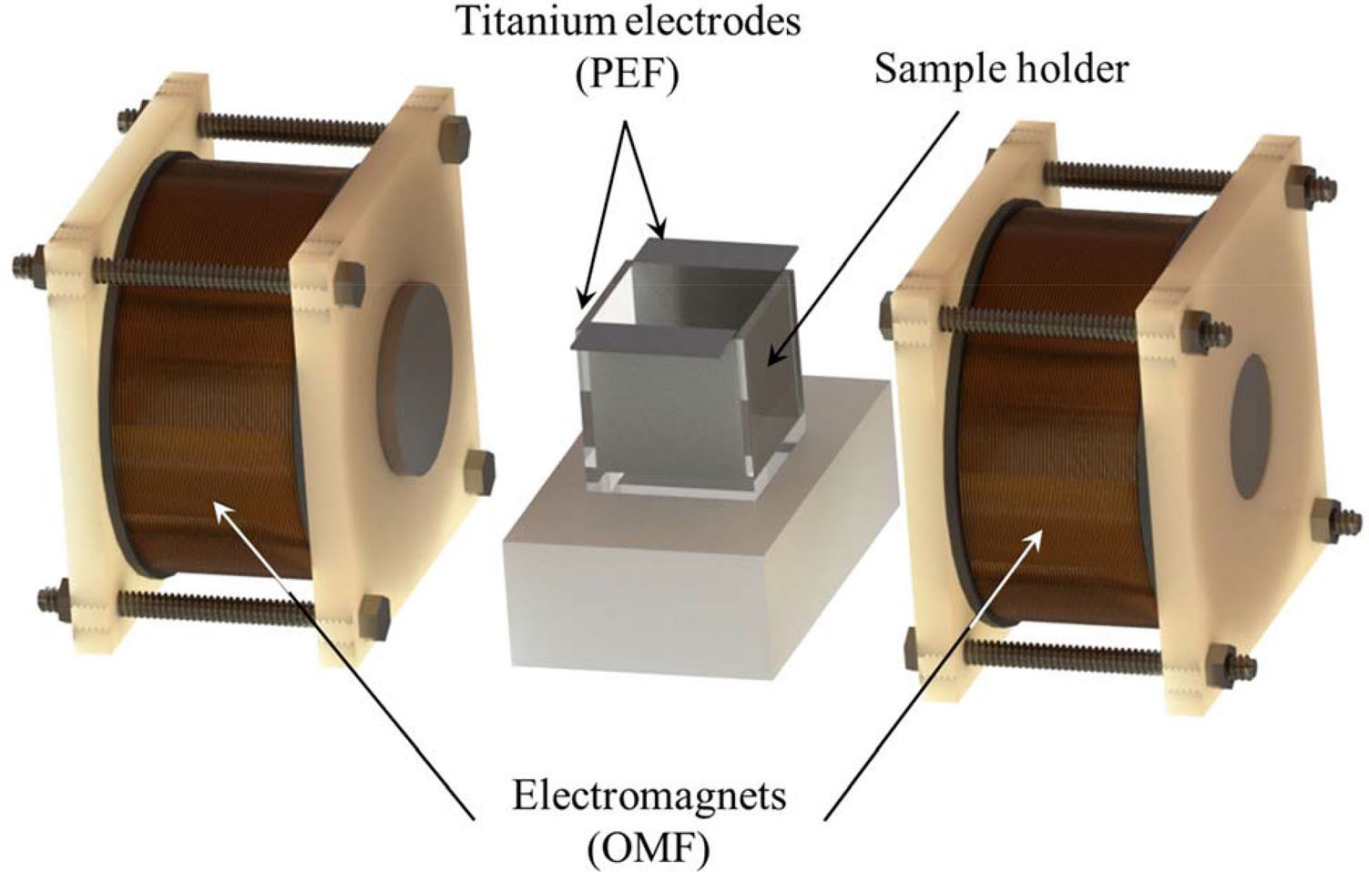
Schematic diagram of experimental set-up for the combination treatment of pulsed electric field (PEF) and oscillating magnetic field (OMF) (You et al., 2020)

On the other hand, innovative and practical approaches have been suggested and examined to achieve stable and acceptable reproducibility of supercooling phenomena. Huang et al. (2018) accomplished the deep supercooling as low as − 20 °C in an aqueous solution for up to 100 days via the surface sealing by oil and alcohol phases. The deeply supercooled samples showed great stability under various disturbances such as vibration, thermal, and ultrasonic treatment. They extended the supercooling technology for human red blood cell preservation at − 16 °C for an extended period of 100 days and demonstrated the significantly higher recovery rates of hemoglobin. The application of high-pressure technology has been investigated for enhancing supercooling of aqueous glycerol solutions (Miyata et al., 2012) and human lung tissues at − 5 °C (Abe et al., 2006). Wan et al. (2018) demonstrated that rat hearts can be preserved at subzero temperatures without ice formation by combining high-pressure treatment. Recently, EMFs have been utilized to promote and extend supercooling. Monzen et al. (2005) developed a supercooling system equipped with an electrostatic field generator for organ preservation and they reported that rat organs such as heart, liver, and kidney were supercooled at around − 4 °C with electrostatic potential ranging from 100 to 500 V. Likewise, Okamoto et al. (2008) reported that rat lungs were successfully preserved for 17 h at subzero temperature of − 2 °C under EF of 3 kV. They mentioned that the static EF had effects on the molecular movement of water and it eventually prevented ice crystal formation. Kato et al. (2012) utilized variable MF treatment for supercooling preservation of rat hearts at − 3 °C for 1 day. The results showed that the supercooled samples showed better hemodynamic and metabolic performance compared that the conventional storage at 4 °C. Her et al. (2019) and Kang et al. (2019) showed that the oscillating MF treatment prevented ice nucleation within fresh-cut honeydew and pineapples and extended the supercooled state for up to 21 days as low as − 7 °C. The quality assessments indicated that the supercooled samples maintained their original qualities without any deterioration such as the color changes and structural damage as shown in Fig. 3. Moreover, Mok et al. (2017) and You et al. (2020) explored the extension of supercooling within chicken breast meat and beef by utilizing the combination treatment of pulsed EF and oscillating MF (Fig. 4). Examples of the supercooling preservation (direct prevention of ice nucleation during freezing) for the extension of shelf-life of foods and biological substances are summarized in Table 1.

**Table 1:** Summary of the application of supercooling for preserving foods and biological materials

Nos.	Type of process	Sample	Supercooling temperature (°C)	Result	References
1	Controlled temperature conditions	Rat liver	− 6	The higher survival rate after transplantation	Berendsen et al. (2014)
2		Rat hepatocytes	− 4	Higher viability	Usta et al. (2013)
3		Rat kidney	− 2 and − 5	Less structural damage	Sultana et al. (2018)
4		Cauliflower	− 8	–	Fuller and Wisniewski (1998)
5		Water	− 3.5	–	Bédécarrats et al. (2010)
6		Garlic	− 6 and − 9	No freezing damage	James et al. (2009)
7		Vegetables	− 6	No appearance deterioration	James et al. (2011)
8		Fish meat	− 5	No structure damage, texture softening	Fukuma et al. (2012)
9		Rice starch gel	− 7.5	Non-homogeneous clusters	Charoenrein and Preechathammawong (2010)
10		Watery Kimchi	− 3	Delayed the growth of microorganisms	Choi et al. (2017)
11		Kimchi starter	− 5	Cold adaptation, shelf-life extension	Choi et al. (2019)
12	Surface sealing	Water, human red blood cell	As low as − 20	Higher recovery rates	Huang et al. (2018)
13	High-pressure	Aqueous glycerol solutions	Homogeneous nucleation temperature	Enhanced supercooling	Miyata et al. (2012)
14		Human lung	As low as − 5	Increased amount of cysteinyl-leukotrienes	Abe et al. (2006)
15		Rat heart	− 4	Reduced metabolism and improved preservation quality	Wan et al. (2018)
16	Electric field (EF)	Rat heart, liver, kidney	− 4	No tissue damage	Monzen et al. (2005)
17		Rat lung	− 2	Less damage, most indicators were equivalent to fresh lungs	Okamoto et al. (2008)
18	Magnetic field (MF) +EF	Chicken breast	− 7	No muscle fiber damage, low drip loss	Mok et al. (2017)
19		Beef	− 4	No ice damage and extended shelf-life	You et al. (2020)
20	MF	Rat heart	− 3	Better hemodynamic and metabolic performance	Kato et al. (2012)
21		Honeydew	− 5.5	No cell damage, lower drip loss, delayed the growth of microorganisms	Her et al. (2019)
22		Pineapple, agar gel	− 7	No cell damage, lower drip loss	Kang et al. (2019)

## Control of ice nucleation using external electric and magnetic fields

### EF and MF for controlled ice nucleation during freezing

Recently, several techniques have been proposed and developed to manipulate the nucleation processes; however, only EMFs have been employed in a commercialized refrigeration system (Rodríguez et al., 2019). The application of EMFs seems more advantageous from the viewpoint of lowering operational costs such as energy consumption and operation simplicity in compassion with other techniques. For example, James et al. (2015a) stated that the capital cost of equipment, the inherent batch nature of the current pressure-assisted freezing are obstacles to the further development of the technology. EMF is a combination of EF and MF that change with space and time. The spatial variation of EMF is dictated by the electromagnetic properties of the material medium such as electrical permittivity and magnetic permeability. There is a direct relationship between the electric flux density (**D**, C/m) and the electric field intensity (**E**, V/m). The relationship between the electric flux density and the electric field intensity is represented as:

12 $${\mathbf{D}} = \varepsilon {\mathbf{E}}$$

where $\varepsilon$ is permittivity of the medium. The permittivity of any medium is defined as:

13 $$\varepsilon = \varepsilon_{0} \varepsilon_{\text{r}}$$

where $\varepsilon_{0}$ is the permittivity of the free space and its value is 8.854 × 10^−12^ F/m, $\varepsilon_{\text{r}}$ is the relative permittivity of the material. The current density of a material is directly related to the electric field intensity according to Ohm’s law. Thus, the current density can be expressed as:

14 $${\text{J}} = \sigma {\mathbf{E}}$$

where ${\text{J}}$ is the current density (A/m^2^), $\sigma$ is the electrical conductivity (S/m). Moreover, it is found that there is a direct relationship between the magnetic flux density (**B**, Wb/m^2^ or Tesla) and the magnetic field intensity (**H**, A/m). The relationship between the magnetic field flux density and magnetic field intensity is defined as:

15 $${\mathbf{B}} = \mu {\mathbf{H}}$$

where $\mu$ is permeability. The permeability of any medium can be expressed as:

16 $$\mu = \mu_{0} \mu_{\text{r}}$$

where $\mu_{0}$ is the permeability of the free space and its value is 4 ${\pi}$ × 10^7^ H/m, $\mu_{\text{r}}$ is the relative permeability of the material. Maxwell’s equations describe the relationship between spatially and temporally averaged EF and MF. Maxwell’s equations can be specified in differential form as following:

17 $$Gauss's\,law\quad \nabla \cdot {\mathbf{E}} = \frac{{\rho_{v} }}{{\varepsilon_{0} }}$$

18 $$Gauss's\,law\,for\,magnetism\quad \nabla \cdot {\mathbf{B}} = 0$$

19 $$Faraday's\,law\quad \nabla \times {\mathbf{E}} = - \frac{{\partial {\mathbf{B}}}}{\partial t}$$

20 $$Ampere - Maxwell \, law\quad \nabla \times {\mathbf{B}} = \mu_{0} {\text{J}} + \mu_{0} \varepsilon_{0} \frac{{\partial {\mathbf{E}}}}{\partial t}$$

Equations 17 and 19 indicate that the sources of EMFs and waves are charges and currents. Moreover, Eq. 18 indicates that magnetic flux lines are always continuous and they form closed loops. It can be seen from Eq. 20 that a time-varying MF generates an EF and Eq. 19 implies that MF can be produced by the flow of current or movement of charges. It is important to note that materials can be treated with either static or time-varying EF and MF. In particular, the EF and MF are interdependent when dealing with time-varying fields, implying that time-varying EF produces time-varying MF and vice versa according to Ampere-Maxwell law and Faraday’s law (Ida, 2015). Thus, the impacts of static and time-varying fields on ice nucleation should be independently investigated and the induced field effects should not be neglected when studying time-varying EF and MF (Kaku et al., 2012; Otero et al. 2016). In recent years, the possible influences of EF and MF on freezing processes have been extensively investigated. It should be noted that most studies have attempted to address a possible association between the fields and water molecules within biological materials and the fundamental basis of the theory of EF effects on nucleation is more obvious than that of MF effects (Rodríguez et al., 2019). In the following sections, theoretical aspects and experimental studies of EF and MF on water and the nucleation processes will be addressed.

## Theoretical aspects of EF on the control of ice nucleation

Water molecules are influenced by EFs due to the intrinsic electric dipole moment and dipole polarizability of the molecules (Woo and Mujumdar, 2010; Wowk, 2012; Xanthakis et al., 2014b). The dipolar moment of water molecules tends to align with the direction of the applied EF vector from random directions and the realignment and reorientation of water molecules result in the redistribution of hydrogen bonds between the molecules (Sastry et al., 2014; Xanthakis et al., 2014a; Zhu et al., 2019). According to the types of applied EF, the EF-assisted freezing can be categorized into two subsections: static electric field (SEF) and alternating electric field (AEF) and theoretical aspects and impacts of SEF and AEF appear clearly different from each other. A static electric field or electrostatic field is a constant field, which doesn’t alter in strength and direction over time. In general, SEF is established by applying a high DC voltage such as several kV between two electrodes that do not directly contact with a sample (Dalvi-Isfahan et al., 2017a). The strength of SEF is expressed in voltages per gap between the electrodes (V/m). The postulated mechanism for the application of SEF during freezing process is associated with the further reduction of nucleation activation energy (Carpenter and Bahadur, 2015) and alignment of water molecules due to the dipole moments of water (Choi et al., 2006; Orlowska et al., 2009), these physical actions enable to induce ice nucleation much easier in supercooled products. In the theoretical approaches of SEF on nucleation, the thermodynamic studies regarding the changes in Gibbs free energy have been frequently carried out (Jha et al., 2017a; Orlowska et al., 2009; Stan et al., 2010). Under an electric field *E,* the new free energy function $G_{E}$ is written by:

21 $$G_{E} = U - TS + pV - V_{C} E \cdot D$$

where $U$ is the internal energy, $T$ is the absolute temperature, $S$ is the entropy, $p$ is the pressure, $V$ is the volume, $V_{C}$ is the volume of the system under the electric field, $D$ is the electric displacement field for a linear system taking permanent polarization *P* (Marand et al., 1988). The electric displacement related to the electric field *E* is defined as:

22 $$D = \varepsilon_{0} \varepsilon_{r} E + P$$

where $\varepsilon_{0}$ is the relative permittivity of the system and $\varepsilon_{r}$ is the permittivity in the vacuum.

The free energy of formation of a spherical nucleus under EF can be rewritten by modifying Eq. 2 (Orlowska et al., 2009):

23 $$\Delta G_{n} = 4\pi r^{2} \sigma - \frac{4}{3}\pi r^{3} \left( {\Delta G_{V} + PE} \right)$$

Moreover, the critical radius ($r^{*}$) and the critical free energy $\Delta G_{n}^{*}$ under the presence of an electric field can be written as:

24 $$r^{*} = \frac{2\sigma }{{\left( {G_{V} + PE} \right)}}$$

25 $$\Delta G_{n}^{*} = \frac{16}{3} \frac{{\pi \sigma^{3} }}{{\left( {\Delta G_{V} + PE} \right)^{2} }}$$

Recently, Jha et al. (2017a) and Zaritzky (2016) have projected a theoretical perspective to evaluate the effect of SEF on the nucleation rate by substituting the free energy terms into relation to the degree of supercooling presented in a substance based upon the classical nucleation theory. The concentration of nuclei in the presence ($$N^{*} )$$ and absence ($$N_{0}^{*} )$$ of EF can be described as:

26 $$N^{*} = N_{1} \exp \left[ {\frac{{ - 16\pi \sigma^{3} }}{{3kT\left( {\frac{{\Delta H_{f} \Delta T}}{{T^{*} }} + PE} \right)^{2} }}} \right]$$

27 $$N_{0}^{*} = N_{1} \exp \left[ {\frac{{ - 16\pi \sigma^{3} }}{{3kT\left( {\frac{{\Delta H_{f} \Delta T}}{{T^{*} }}} \right)^{2} }}} \right]$$

In order to express the concentrations of the nuclei as a function of electric field strength, Eq. 26 can be divided by Eq. 27 and then the nucleation induction period ($\tau )$ can be described as (Jha et al., 2017a; Nagalingam et al., 1981):

28 $$\frac{1}{\tau } \propto { \log }_{10} \left( {\frac{{N^{*} }}{{N_{0}^{*} }}} \right) = \left( {\frac{{2.32\pi \sigma^{3} }}{kT}} \right)\left[ {\left( {\frac{1}{{\frac{{\Delta H_{f} \Delta T}}{{T^{*} }}}}} \right)^{2} - \left( {\frac{1}{{\frac{{\Delta H_{f} \Delta T}}{{T^{*} }} + PE}}} \right)^{2} } \right]$$

**Fig. 5:**
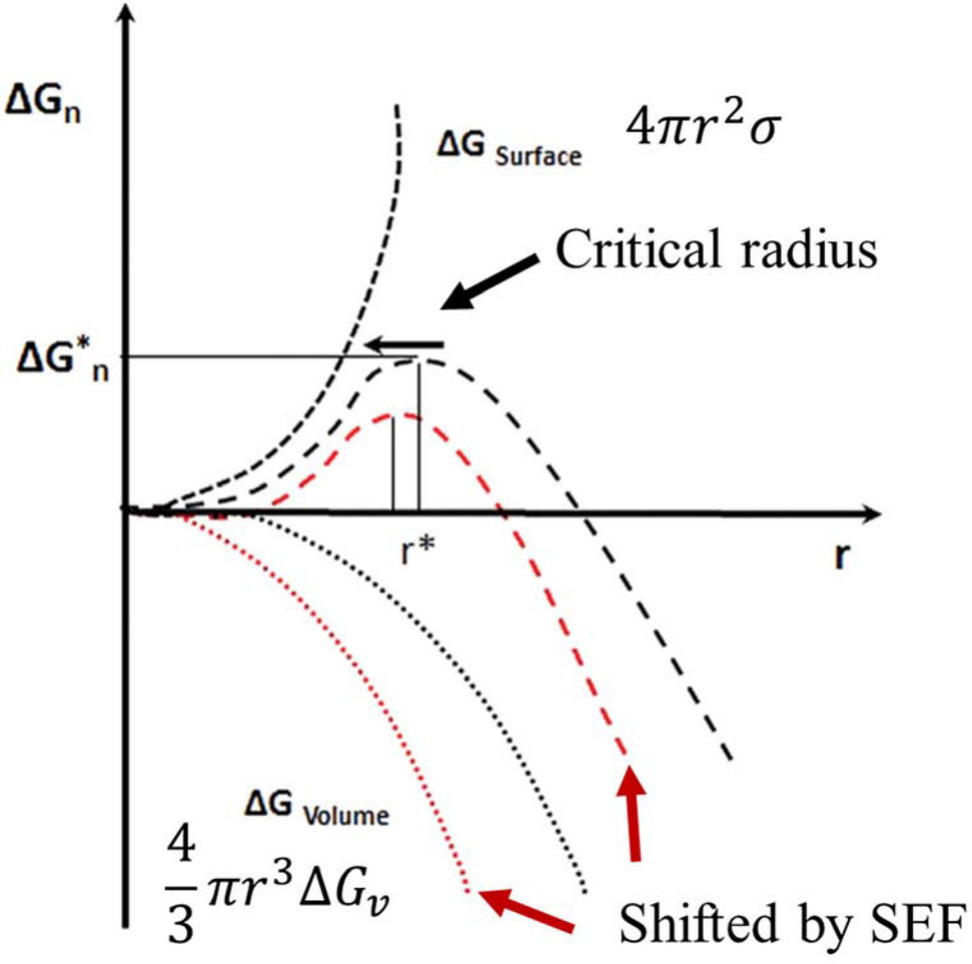
Schematic diagram of changes in Gibbs free energy and the critical nucleus radius by an electrostatic field during freezing process of water. Modified from Dalvi-Isfahan et al. (2017b)

From a thermodynamic point of view, it is seen that the application of SEF allows modifying the free energy potentials and the size of critical ice embryo in a supercooled liquid. As a result, the rate of ice nucleation becomes noticeably accelerated and the supercooled state will be suppressed during freezing (promotion of ice nucleation) by SEF treatment (Dalvi-Isfahan et al., 2017a; Jha et al., 2017a). In a recently published review paper, the changes in Gibbs free energy and critical radius by applying an external electrostatic field have been well described as shown in Fig. 5 (Dalvi-Isfahan et al., 2017b). Unlike SEF, AEF with at least a radio frequency band might inhibit ice nucleation in supercooled water and consequently increase the degree of supercooling during freezing (Dalvi-Isfahan et al. 2017b). An electric field produced by alternating currents is called an oscillating electric field, an AC electric field, or an alternated electric field depending on authors and contexts (Sun et al., 2006c; Woo and Mujumdar, 2010; Wowk, 2012). Although it has not been fully confirmed yet, the predominant mechanism for the inhibition of ice nucleation is that AEF exerts a torque that can displace, disturb, and/or rotate water molecules in response to the field (Cheng et al., 2017; Woo and Mujumdar, 2010).

## Experimental studies: the effects of SEF on freezing

SEF has been utilized to modify the freezing process in water and biological materials and shown several measurable effects; however, it is likely that the effective strengths of SEF vary. According to the literature, SEF above 10^9^ V/m strength certainly changes the structure of water (Sun et al., 2006a; Svishchev and Kusalik, 1994) and some physicochemical properties as well (Jung et al., 1999; Shevkunov and Vegiri, 2002). Meanwhile, Stan et al. (2010) suggested that the range of 10^7^–10^8^ V/m might cause an increase in the rate of nucleation based upon thermodynamic models; however, SEF with amplitudes up to 1.6 × 10^6^ V/m neither enhanced nor suppress nucleation. Carpenter and Bahadur (2015) also found that SEF treatment (> 2.0 × 10^7^ V/m) elevated the nucleation temperature of water. In contrast, Orlowska et al. (2009) showed that the significant changes in the degree of supercooling within water were observed in a ranging from 0 V to 6.0 × 10^6^ V/m, but further increase of SMF strength was only marginal. Furthermore, the promoted ice nucleation was also found under SEF at a strength of 1.0 × 10^5^ V/m (Wei et al., 2008). In food freezing process, SEF has been exploited to improve the final quality of the frozen products by inducing nucleation, reducing the size of ice crystals, and decreasing the phase-transition time (Dalvi-Isfahan et al., 2017a; Fallah-Joshaqani et al., 2019; Jia et al., 2017). For instance, SEF treatments on pork tenderloin samples during the freezing process have shown smaller ice crystal formations with increasing the strength of SEF. The SEF with a strength of 12 kV significantly reduced the degree of supercooling within pork by reorientation water molecules along the SMF (Xanthakis et al., 2013). Similar results have been reported by Dalvi-Isfahan et al. (2016). The authors froze lamb sirloin under SEF up to 5.8 × 10^4^ V/m and compared the quality with the conventional freezing process. The results showed that the size of ice crystals in the meat under the SEF decreased and the improved drip loss and texture properties. In the current state of knowledge, SEF is considered as a promising tool in food freezing industry because it effectively suppresses supercooling and reduces the phase transition time with negligibly small or little power consumption (Dalvi-Isfahan et al., 2017b; Jia et al., 2017). However, it is not clear how feasible it will be to integrate SEF modules into commercial freezing compartments (James et al., 2015b).

## Experimental studies: the effects of AEF on freezing

Analogous to SMF application, AEF can be generated between electrodes that do not come into direct contact with a sample. Anese et al. (2012) reported that the radiofrequency pulses with different voltages such as 2 kV and 7 kV allowed to induce water dipole rotation, which resulted in the reduced size of ice crystals within pork meat. In like manner, radiofrequency waves were used to rotate and mobilize water molecules in fish fillets. The results showed that the rotation of water molecules led to a higher degree of supercooling (Hafezparast-Moadab et al., 2018). In the claimed patent, AEF generated by a radio frequency voltage prevented freezing of foods at above − 5 °C (minimum allowable supercooling temperature). It is likely that the applied AEF continuously vibrated, rotated, and translated water molecules due to the polarity of the molecules and these actions maintained the supercooled state of water without crystallization (Kim et al., 2013b). In this sense, Ma et al. (2013) confirmed that the degree of supercooling in 0.9% NaCl aqueous solution was enhanced by an AC electric field (100 kV/m at 10^6^ Hz) due to the induced electric dipole oscillation. Conversely, the physical mechanisms for AEF studies involving in direct contact with electrodes are more complex and include different effects such as the effect of an electric field, electric current, and air bubbles, etc. For example, pulsed AEF (1.78 × 10^2^ V/m at 20 kHz) in direct contact with a sample was successfully applied to control the freezing behaviors of 0.9% NaCl solution, in particular for the formation of uniform and small ice crystals and reduction of the phase transition time (Mok et al., 2015). By extension, the authors tested the AEF for the prevention of sudden ice nucleation in a real food system while combining OMF. The electrical current passing through the sample was measured to be 32 mA at the maximum (Mok et al., 2017). Sun et al. (2006c) showed that AEF with a wide range of frequencies (50 Hz to 5 MHz) directly acts on the nucleation process of 0.9% NaCl solution. Remarkably, the AEF with 500 kHz led to the minimum degree of supercooling, while the increased supercooling was observed at a frequency of 5 MHz. A similar investigation was conducted by the same research group. Sun et al. (2006b)showed the impact of AEF on the ice formation in 0.9% K_2_MnO_4_ water. They applied AEF with frequencies ranging from 1 to 200 kHz during the freezing process and observed the ice morphology and freezing time. The results indicated that the polarization induced by AEF induced the reorientation of water molecules and disturbed the aggregation of the molecules. The size of ice crystal and freezing time were decreased by the increased frequency and reached the minimum at the frequency of 50 kHz, which was close to the frequency at which the dielectric relaxation peak of ice appears. Furthermore, Ninagawa et al. (2016) showed that a micro-electric current below 10 µA significantly changed the intracellular ice crystal behaviors while increasing supercooling degree and minimizing the cell deformation. On the other hand, delivering direct current to a sample seems to induce ice nucleation (Okawa et al., 2011; Pruppacher et al., 1968; Shichiri and Nagata, 1981). Specifically, Carpenter and Bahadur (2015) presented the possible mechanisms involved in ice nucleation under electrowetting fields. They stated that the electric current flow can trigger heterogeneous nucleation due to the generation of bubbles as a result of electrolysis and other chemical reactions at the surface of the electrode. Hozumi et al. (2003, 2005) reported that the small electrical current within 5 µA (DC voltage applied) could induce nucleation in supercooled water and the probability of ice nucleation was varied depending upon the material types and shapes of electrodes due to the surface properties and the degree of ionization.

## Theoretical aspects of MF on the control of ice nucleation

All matters exhibit magnetic effects since the electrons in atoms spin and create tiny magnetic fields. Magnetic properties of materials can be classified as diamagnetic, paramagnetic, or ferromagnetic. Water is a typical diamagnetic material, indicating that water has no net magnetic moment in the absence of an external MF since the orbital magnetic moment and the electron spin magnetic moment cancel each other. Under MF, the orbital motion of electrons is changed and a small magnetic moment is induced in the opposite direction of the applied MF in compliance with the Lenz’ law (Otero et al., 2016). The magnetic force exerted on a diamagnetic material is proportional to the intensity and gradient of MF and the magnetic susceptibility, implying that water will become weakly magnetized by a weak MF due to its low magnetic susceptibility (χ = − 9.07 × 10^−9^ m^3^/kg at 20 °C) (Jha et al. 2017a). On the other hand, a very strong MF (> 10 T) can exert influential force on water and it can be macroscopically visualized such as levitation against the gravity (Beaugnon and Tournier, 1991; Ikezoe et al., 1998) and the physical deformations (Chen and Dahlberg, 2011; Kitazawa et al., 2001; Pang and Deng, 2008).

Research on the effects of MF on water has been widely performed using computational simulation techniques and experimental validation. Computer simulation techniques have been employed to investigate the structure and dynamic behavior of water under an external MF. Chang and Weng (2006) investigated the effects of static MF with intensities ranging from 1 to 10 T on the structural changes of liquid water using molecular dynamics simulation. The results showed that the self-diffusion coefficient of water was reduced and the number of hydrogen bonds was slightly increased (approximately 0.34%) when exposed to MF. In other words, SMF enhances the hydrogen-bonding network by forming a tighter bonding between molecules, which constrains the movement of the water molecules in the liquid state. Zhou et al. (2000) carried out a Monte Carlo simulation to determine the influence of an external MF on the internal energy and heat capacity of pure water. The authors found that the application of a magnetic flux density (200 mT) significantly changed the thermal properties of water; whereas 5 mT had no effects on the structure of water. They mentioned that the structural change of water and magnetic moment interaction due to the exposure of magnetic fields will strongly affect the hydrogen bond distribution and the internal energy of water. In addition, the external MF may weaken and partly break hydrogen bonds while increasing the number of monomer water molecules. Experimental data have shown that MF treatments significantly influenced microscopic structures and macroscopic properties of water. Iwasaka and Ueno (1998) utilized a 14 T superconducting magnet to study the structure of water molecules and suggested a possibility that the application of the magnetic field affected the formation of hydrogen bonds of water molecules. Inaba et al. (2004) showed that the melting point of water was proportionally increased with increasing the intensity of applied magnetic fields ranging from 4 to 6 T. They suggested that the shift of the melting temperature was derived from the strengthened hydrogen bonding between water molecules. Pang and Deng (2008) observed the changes in the spectrum shape, the tendency of fluid surface, rheology, refraction index, and electrical properties of water under MF and concluded that that the application of a static MF (440 mT) caused the displacements and polarization of molecules and atoms, leading to the changes of distribution of molecules and electrons. Furthermore, the MF treatment affected the dipole-moment transition and vibrational states of water molecules. Similarly, Cai et al. (2009) studied the effects of a static MF (500 mT) on the changes in physicochemical properties of water. The authors suggested that the static MF treatment had effects on the intramolecular energy and the activation energy of water. Moreover, the rotational motion of water molecules was significantly decreased under MF, which indicated that more hydrogen bonds were formed and the average size of water clusters was increased by SMF treatment. Wang et al. (2013) examined the effect of static MFs (270 and 530 mT) on hydrogen bonding interactions between water molecules. The results showed that lower energy dissipation was found in the magnetized water and the friction coefficient was decreased with increasing the magnetic field intensity. They suggested that the interaction of SMF based upon Lorentz forces can weaken or break hydrogen bonding in water. Interestingly, similar findings have been reported in relatively weak MFs. Toledo et al. (2008) examined the influences of much weak static MFs (45–65 mT) on the physicochemical properties of water. They suggested that the MF treatment weakened the intra-cluster hydrogen bonds by breaking the large water clusters and forming the small water clusters, which stabilized inter-cluster hydrogen bonds. Furthermore, Szczes et al. (2011) reported that the magnetized water by a weak MF (15 mT) showed the meaningfully higher conductivity and the evaporation rate during circulation of water, this may be derived from the strengthened hydrogen bond network and the perturbation of gas/liquid interface from the air nanobubbles in the water. From the literature, it is clear that an external MF influences water and the predominant mechanism suggested by different authors is associated with the hydrogen bonds. In most cases, MFs can either be static (SMF) or oscillating (OMF). SMF is most likely to be generated by permanent magnets with different configurations and sizes and OMF is mainly achieved with different types of electromagnets such as a ferric core-based electromagnet or Helmholtz coils.

## Experimental studies: the effects of static MF on freezing

There are several research papers that described the effects of a static magnetic field (SMF) on ice nucleation and crystallization processes during freezing. However, unfortunately, experimental data in the literature have appeared to be contradictory and showed low reproducibility. Currently, the accurate mechanisms have not been completely elucidated, but the majority of studies suggest that hydrogen bonds between water molecules become strengthened under SMF and the molecules and/or water clusters are more ordered and stably distributed, therefore, ice nucleation is promoted (Cheng et al., 2017; Woo and Mujumdar, 2010). For example, Aleksandrov et al. (2000) found that supercooling of water drops decreased with SMF at an intensity of 71 mT and the degree was decreased with increasing magnetic field intensity. Furthermore, supercooling was inconsequential at an intensity of 505 mT. The authors suggested that the diamagnetic effect of SMF affected the orientation ordering of nuclei and induced the coagulation of water molecules. In this sense, Zhao et al. (2017) found that SMF with intensities less than 50 mT caused the significant increase of nucleation temperature for 0.9% NaCl and 5% ethylene glycol solutions; however, no significant differences were found in deionized water. In contrast, stronger SMF ranging from 107–359 mT and 0–241 mT did not show any effects on nucleation, the extension of supercooling, and the phase transition time of 10 mL pure water and 0.9% NaCl solution (Otero et al., 2018). In a recent study, SMF can be combined with other EMFs such as pulsed electric fields for the effective and synergistic freezing process. The authors proposed that the combined SMF (0–480 mT) and pulsed EF reduced the phase transition time and enabled to form the uniform ice crystals in 2 mL of 0.9% NaCl solution due to the distortion of hydrogen bonds (Mok et al., 2015).

According to the literature, SMF has been applied for not only simple matrices but only some complex biological materials. Lin et al. (2013a) tested the survival rate of frozen erythrocytes exposed to SMF with a magnetic induction of 200 mT and 400 mT under the slow freezing at a rate of -1 °C/min. The results indicated that the relative survival rates of samples exposed to SMF were increased without any negative effect on the cell morphology and function. They concluded that a greater effect could be expected if samples are treated with a stronger SMF. Similar results have been reported by Lin et al. (2015). Human dental pulp stem cells were preserved at − 196 °C with SMF with at intensities of 400 mT and 800 mT and the significant increase in the survival rates was found with the SMF treatments. They concluded that SMF may improve cell membrane stability, resulting in less damage caused by ice crystals during the freezing process. In a recent study, the effect of SMF (0–16 mT) and OMF (0–1.8 mT) on the freezing behavior of pork meat has been published from a research group. The results indicated that while SMF significantly decreased the nucleation point, phase transition time, and supercooling time and increased the time through − 1 to − 5 °C; the OMF treatment did not significantly affect the freezing parameters (Tang et al., 2019).

## Experimental studies: the effects of oscillating MF on freezing

In short, the main mechanism for OMF-assisted freezing in the literature is that OMF directly act upon water molecules by re-orientating and vibrating the molecules and/or break hydrogen bonds between water molecules, resulting in a large degree of supercooling and small ice crystal formation within biological materials such as animal tissues, foods, and living cells (Jha et al., 2017b; Otero et al., 2016; Woo and Mujumdar, 2010). However, the mechanism has not been verified in a thorough manner and it still remains a debatable issue. Although there exists a controversy regarding the effects of OMF, some of the experiments have determined the potential role of OMF in the control of ice nucleation and shown interesting results over the past decade. Zhan et al. (2019) showed that OMF with an intensity of 10.0 mT at 100–250 Hz improved the degree of supercooling of two aqueous solutions (sodium chloride and poly dimethyl ammonium chloride) by disturbing the energy balance in solutions. They also reported that the OMF significantly changed the physicochemical properties of the solutions such as pH, surface tension, and contact angle. Liu et al. (2017) explored the effect of OMF (0–7.2 mT at 50 Hz) on the freezing characteristics of carrot strips by observing the ice crystal formation during the freezing process using the optical microscope. They found that the increase in the OMF’s intensity decreased the phase transition time and reduced physical damage to the vegetable. The similar intensity of OMF (8.0 mT at 1 Hz) was utilized to inhibit ice nucleation within honeydew during the supercooling preservation. The authors argued that the OMF prevent nucleation and extended the supercooled state of honeydew cubes at around − 5.5 °C (Her et al., 2019). The magnetic flux density of OMF seems to be enhanced by combining SMF as demonstrated by Mok et al. (2017). The authors used a block of NdFeB permanent magnet and an electromagnet to produce OMF intensities from 50 to 100 mT. The results showed that the applied OMF inhibited sudden ice nucleation within chicken breast meat during supercooling at the freezing temperature of − 7 °C. Semikhina and Kiselev (1988) reported that bi-distilled water exposed to OMF (0.88 mT at up to 200 Hz) showed a larger degree of supercooling; however, Naito et al. (2012) reported that OMF (0.5 mT at 30 Hz) did not influence on the freezing behavior of distilled water and saline solution. Meanwhile, different viewpoints on the impact of OMF have been introduced. Rodríguez et al. (2019) stated that any variable magnetic fields will induce variable non-conservative electric fields according to Faraday’s law. This fact indicates that the induced electric fields can be associated with the effects that are supposed to be explained by the influence of OMF. It is generally known that electric fields can induce the reorientation and vibration of water since water molecules have dipole moments. In this respect, Kaku et al. (2012) responded to the questions asked by a third party while declaring that OMF (0.1 mT at 60 Hz) induced electric fields that actually inhibit ice crystal formation. Thus, Otero et al. (2016) have stated that the induced electric fields’ effect should be investigated in case there exists OMF. It is likely that the employment of OMF would be an important technological advance to apply oscillating electric fields instead of the use of electrodes (Rodríguez et al. 2019). Apart from that, Kobayashi et al. (2018) offered a feasible point of view for the inhibition of heterogeneous nucleation in supercooled water by OMF treatment. The authors suggested that OMF (1 mT at 10 Hz) induced the magneto-mechanical rotation of the nanophase magnetite, which is naturally presented in biological materials. The physical exercise may disrupt the water–ice crystal interface and suppress heterogeneous ice nucleation. The suggested mechanism seems not to be limited to pure water but, extended to real food matrices such as celery and beef.

## Patented MF-assisted freezing technologies for controlled ice nucleation

Over the past few years, the beneficial effects of OMF on freezing of food and biomaterials have been claimed by a few investigators and manufacturers (Hirasawa et al., 2001; Owada, 2007; Owada and Kurita, 2001; Sato and Fujita, 2008). The intensities of OMF ranges from 0.1 mT and 800 mT in the patents; however, it seems that OMF with intensities less than 1 mT has been commonly applied for a commercialized novel freezing system such as Cell Alive System (CAS, ABI Corporation Ltd, Chiba, Japan). According to the patents invented by CAS, the freezing system equipped with OMF generators is supposed to inhibit ice nucleation and consequently improve the freezing process of diverse biological substances. For instance, Owada (2007) and Owada and Saito (2010) claimed that OMF (less than 1 mT and 50 Hz) reduced the total freezing time and no cell damage was found in frozen-thawed chicken and tuna meat in comparison with fresh food samples. In this way, a few studies have shown significant impacts of OMF-assisted freezing. The applications of OMF (1.5–2.0 mT at 30 Hz) showed advantageous results in the quality of frozen chicken breasts over conventionally frozen samples during 6 months of storage (Yamamoto et al., 2005). Lin et al. (2013b) showed the potential application of a programmed freezing protocol with an OMF for an efficient method of human embryonic stem cell preservation. Compared to other studies, a much weaker OMF intensity (0.01 mT at 60 Hz) also inhibited freezing-induced damage of the cell cytoskeleton, resulting in the prevention of frostbite in mouse legs (Koizumi et al., 2017). Choi et al. (2015) found that the OMF-assisted freezing reduced the ice crystal size in beef steaks and improved sensory characteristics. Similarly, Kim et al. (2013a) argued that the electromagnetic freezing significantly reduced total freezing time and drip loss within beef, pork, and chicken meats. However, Otero et al. (2016) cast doubt on the different storage temperatures in the review paper. In addition, to the best of the author’s knowledge, the number of replications and reproducibility are not clearly mentioned, which seems very important in comparing results between OMF-assisted freezing versus the air blast freezing (controls). As stated, the mechanisms and impacts of OMF-assisted freezing are highly controversial issues and unfortunately, the majority of researchers do not agree with the patent claims. Recently, diverse food materials such as crab sticks (Otero et al., 2017), pork (Rodríguez et al., 2017), apple and potato (Purnell et al., 2017), garlic bulbs (James et al., 2015b), egg yolk and egg white (Fernández-Martín et al., 2017; 2018) have been tested by different authors. The results showed that OMF had no significant effect on the freezing characteristics, the degree of supercooling, and the physicochemical properties of foods after thawing. The authors have cast doubt on the impacts of OMF on water due to its low magnetic susceptibility. On the basis of the results, they have suggested investigating further research at wide ranges of magnetic field intensity and frequency (Otero et al., 2017; Rodríguez et al., 2017). In addition to the OMF’s operation parameters, the effects of OMF can vary depending upon cooling rate, storage temperature, and storage period and it may not affect all biological samples in an equal manner (Purnell et al., 2017). Particularly, the inherent discrepancy of biomaterials such as size, shape, structure, variety, and composition also contribute to diffuse the OMF effects (Otero et al., 2017). Therefore, intensive research is still required to establish and verify the potential role of OMF in food freezing and supercooling.

**Fig. 6:**
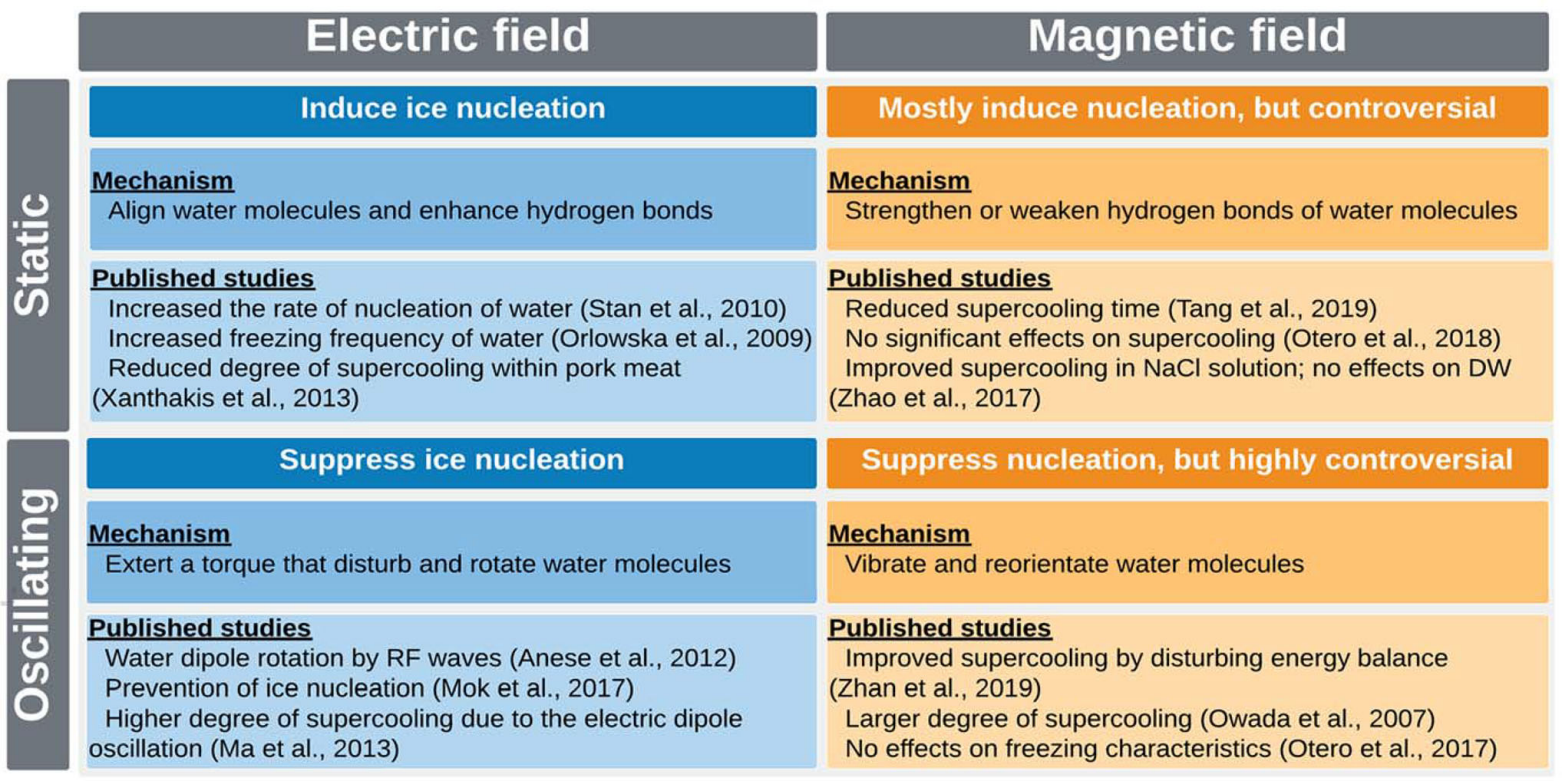
Summary of the effects, mechanisms, and key published studies on electric and magnetic fields-assisted controlled ice nucleation during freezing

In conclusion, supercooling preservation has great potential to maintain food quality and extend the storage life of biological materials; however, the current state of the technology has not matured enough to be widely adopted in commercial applications. In spite of the fact that ice nucleation is one of the most important factors in conventional freezing processes and the supercooling preservation, it remains an uncontrolled variable due to the complexities of the phenomenon and the stochastic nature. The application of electric and magnetic fields has been shown the potential to control ice nucleation within foods and biomaterials. In summary, SEF and SMF are potentially able to induce ice nucleation in a supercooled product and AEF and OMF tend to exert the opposite effects. However, some of the mechanisms and detailed effects have not adequately elucidated yet and results in the literature appear contradictory, in particular, AEF and OMF’s impacts (summarized in Fig. 6). To shed light on the role of electric and magnetic fields on supercooling, a wide range of intensities and frequencies of the fields should be investigated. Moreover, proper system design and the practical operating conditions for the EMF application should be taken into consideration when integrating into existing freezing systems.

## List of symbols

A: Ampere
AC: Alternating current
AEF: Alternating electric field
**B**: Magnetic flux density
${\text{C}}_{0}$: Concentration of nucleation sites
CAS: Cell alive system
CNT: Classical nucleation theory
**D**: Electric flux density
$$D$$: Electric displacement
DC: Direct current
**E**: Electric field intensity
EF: Electric field
EMF: Electromagnetic field
*f*^***^: Frequency of monomer attachment to nucleus
$\Delta G_{n}$: Gibbs free energy of a new phase
$\Delta G_{n}^{*}$: Critical Gibbs free energy of a new phase
$\Delta G_{S}$: Surface free energy of the nuclei
$\Delta G_{V}$: Volumetric free energy of the nuclei
**H**: Magnetic field intensity
$\Delta H_{f}$: Enthalpy of fusion
Hz: Hertz
**J**: Current density
$$J$$: Nucleation rate
**J**^e^: Externally generated current density
$$k_{B}$$: Boltzmann constant
MF: Magnetic field
$N$: Number of ice nuclei per unit volume
*N*^*^: Concentration of nuclei
OMF: Oscillating magnetic field
*P*: Permanent polarization
*r*: Radius
$$r^{*}$$: Critical radius
*S*: Entropy
SEF: Static electric field
SMF: Static magnetic field
T: Tesla
*T*: Temperature
$\Delta T$: Degree of supercooling
$$T_{m}$$: Melting point
$$U$$: Internal energy
V: Voltage
*V*: Volume
*Z*: Zeldovich factor
$\varepsilon$: Permittivity of the medium
$\varepsilon_{0}$: Permittivity of the free space
$$\uptheta$$: Wetting angle
*μ*: Permeability
$\mu_{0}$: Permeability of the free space
$$\mu_{\text{r}}$$: Relative permeability
$\rho_{v}$: Electric charge density
σ: Surface energy of the particle per unit volume
$$\sigma \left( {\text{S/m}} \right)$$: Electrical conductivity
$\tau$: Nucleation induction period
χ: Magnetic susceptibility
