# Correction to: Supercooling preservation technology in food and biological samples: a review focused on electric and magnetic field applications

**DOI:** 10.1007/s10068-020-00786-8

**Published:** 2020-07-08

**Authors:** Taiyoung Kang, Youngsang You, Soojin Jun

**Affiliations:** 1 Department of Molecular Biosciences and Bioengineering, University of Hawaii at Manoa, Honolulu, HI 96822 USA; 2 Department of Human Nutrition, Food and Animal Sciences, University of Hawaii at Manoa, Honolulu, HI 96822 USA

## Food Sci Biotechnol (2020) 29(3):303–321 10.1007/s10068-020-00750-6

The article “Supercooling preservation technology in food and biological samples: a review focused on electric and magnetic field applications”, written by Taiyoung Kang, Youngsang You and Soojin Jun, was originally published Online First without Open Access. After publication in volume 29, issue 3, pages 303–321 the author decided to opt for Open Choice and to make the article an Open Access publication. Therefore, the copyright of the article has been changed to © The Author(s) 2020 and the article is forthwith distributed under the terms of the Creative Commons Attribution 4.0 International License (http://creativecommons.org/licenses/by/4.0/), which permits use, duplication, adaptation, distribution and reproduction in any medium or format, as long as you give appropriate credit to the original author(s) and the source, provide a link to the Creative Commons license, and indicate if changes were made.

The original article has been corrected.

